# Quantitative magnetic resonance imaging parameters of lumbar paraspinal muscle impairment in myotonic dystrophy type 2 and their evolution with aging

**DOI:** 10.3389/fneur.2025.1525952

**Published:** 2025-02-19

**Authors:** Viktoria Kokosova, Peter Krkoska, Daniela Vlazna, Michaela Sladeckova, Marek Dostal, Milos Kerkovsky, Tamara Barusova, Petra Ovesna, Olesja Parmova, Katerina Matulova, Blanka Adamova

**Affiliations:** ^1^Department of Neurology, Centre for Neuromuscular Diseases (Associated National Centre in the ERN EURO-NMD), University Hospital Brno, Brno, Czechia; ^2^Faculty of Medicine, Masaryk University, Brno, Czechia; ^3^Department of Rehabilitation, University Hospital Brno, Brno, Czechia; ^4^Department of Public Health, Faculty of Medicine, Masaryk University, Brno, Czechia; ^5^Department of Radiology and Nuclear Medicine, University Hospital Brno, Brno, Czechia; ^6^Institute of Biostatistics and Analyses Ltd., Brno, Czechia

**Keywords:** myotonic dystrophy type 2, magnetic resonace imaging, muscle strengh, endurance, paraspinal muscles

## Abstract

**Introduction:**

Muscle magnetic resonance imaging (MRI) is an emerging method in the diagnosis and monitoring of muscular dystrophies. This cross-sectional, comparative study aimed to evaluate quantitative MRI (qMRI) parameters of the lumbar paraspinal muscles (LPM) in myotonic dystrophy type 2 (DM2), to assess their relationship with functional examination, and to evaluate their evolution with aging.

**Methods:**

The study enrolled 37 DM2 patients and 90 healthy volunteers (HV) who were matched based on physiological parameters to create 35 pairs. Utilizing a 6-point Dixon gradient echo sequence MRI, fat fraction (FF), total muscle volume, and functional muscle volume (FMV) of the LPM and psoas muscle (PS) were obtained. Using correlation coefficients and regression models, the relationship between MRI and the maximal isometric lumbar extensor muscle strength (MILEMS) and lumbar extensor muscle endurance (LEME), and their evolution with age, were assessed.

**Results:**

LPM showed significantly higher FF in DM2 patients compared to HV (21.3% vs. 11.3%, *p*-value <0.001). FMV of LPM correlated significantly with MILEMS (*ρ* = 0.5, *p*- value = 0.001) and FF with LEME (*ρ* = −0.49, *p*- value = 0.002) in DM2. No significant differences in the rate of deterioration in functional and morphological parameters of the LPM with age were observed between the two groups.

**Conclusion:**

We demonstrated morphological correlates of lumbar extensor muscle dysfunction in DM2 patients. The qMRI parameters of LPM correlated with functional parameters but could not be used either as a reliable biomarker of lumbar extensor muscle impairment or as a biomarker of disease progression.

## 1 Introduction

Myotonic dystrophy type 2 (DM2) is an autosomal dominant hereditary multisystem disease that is characterized by myotonia, muscle weakness, and early-onset cataracts (before the age of 50) (1). It is the most common adult-onset muscular dystrophy in Central and Northern Europe (Finland) with an estimated prevalence of about 9 in 100,000 (2). Patients with DM2 typically experience weakness in the proximal limb muscles, with a proximo-distal pattern of progression (3). Truncal (axial) muscles are also affected. Thus, DM2 can be classified as an axial myopathy with significant paraspinal involvement as part of a more widespread myopathy (4). Examination of the paraspinal muscles (both clinical and MRI) in patients with DM2 is often unjustly neglected, although proper functioning of these muscles is essential. These postural muscles stabilize the spine and play a crucial role in daily mobility and spinal health (5, 6). Significant dysfunction of the lumbar extensor muscles (LEM), which include the lumbar paraspinal muscles (LPM), in DM2 was shown in previous study. Reduced LEM strength was an independent risk factor of the frequent occurrence of chronic low back pain in these patients (7).

Magnetic resonance imaging (MRI) of muscles has become a very important tool in diagnosing and monitoring disease progression and treating neuromuscular disorders including myopathies (8, 9). In general, there has been an effort to identify MRI biological markers (biomarkers) that would be objective indicators of the muscle structure, reflect pathological processes in the muscle, and determine the severity of the disease. Currently, the percentage of intramuscular fat (fat fraction – FF), the muscle cross-sectional area or total muscle volume (TMV) and functional muscle volume (FMV), that is easily derived from TMV and FF (10), are considered as qMRI (quantitative MRI) muscle biomarkers, with FF and FMV as key biomarkers (11).

Few studies have evaluated muscle MRI findings in patients with DM2 (12–15) and only one study evaluated the qMRI parameters of lower limb muscles in DM2 patients (12). Previous reports of muscle MRI in DM2 suggest that the erector spinae, gluteus maximus muscle, and thigh muscles (slightly more pronounced in the posterior compartment) are primarily affected by increased fat replacement of muscle tissues (12–15). However, it has been documented that patients with DM2 frequently exhibit normal muscle MRI findings, with muscle fat infiltration only becoming evident in the late stages of the disease (13, 14). When assessing the morphology of the LPM, it is important to remember that it is influenced by several physiological variables, mostly by age, sex, and anthropometric parameters (16–21).

An unresolved issue is the correlation between morphological MRI parameters and muscle function. It has been reported that the performance of muscles is largely dependent on muscle mass and muscle composition (10).

The aims of this study were; (1) to evaluate qMRI parameters of the LPM in patients with DM2 and compare them to healthy volunteers (HV); (2) to explore the relation between qMRI parameters of LPM and functional LEM parameters in DM2; and (3) to evaluate whether dysfunction and morphological parameters of LPM in patients with DM2 additionally worsen with age compared to HV, suggesting progressive impairment in these muscles. The results of this study will help to determine whether qMRI parameters of LPM can be reliable biomarkers of LEM dysfunction and disease progression in DM2.

## 2 Methods

This was a single-center, observational, cross-sectional, comparative study. The study protocol (agreement number 05-090621/EK) was approved by the local institutional medical research ethics committee, and all participants provided written informed consent.

### 2.1 Participants

The study involved patients with DM2 and HV.

Patients with DM2 were recruited from a cohort of patients with a genetically confirmed diagnosis of DM2 who were enrolled in the registry of muscular dystrophies (REaDY) and had a neurological check-up between May 2021 and March 2023. HV were recruited from a control database that was developed for a long-term project investigating the function and morphology of LPM at our neuromuscular center. The inclusion criteria were age ≥ 18 years. Exclusion criteria were general MRI contraindications, and the presence of metal material in the lumbar spine, even MRI-compatible metal, as artifacts can influence measurement accuracy, paresis of the hip extension evaluated by manual muscle testing and defined by the Medical Research Council (MRC) scale less than grade 4-, previous lumbar spine involvement (vertebral fracture, tumor, spine infection), comorbid conditions affecting the overall mobility of the patient (e.g., post-stroke paresis, heart failure leading to limited mobility), confirmed pregnancy, and significant impairment of cognitive functions. For HV, additional exclusion criteria were medical history of chronic low back pain (of a duration over 12 weeks) or lumbosacral radicular pain with residual signs of nerve root dysfunction in clinical neurological examination, previous surgery of the lumbar spine, acute low back pain, scoliosis, degenerative changes in the lumbar spine (the presence of lumbar spinal stenosis - Schizas grading scale above A4) and lumbar disk herniation.

### 2.2 Procedures

#### 2.2.1 Medical history and clinical examination

All subjects (patients with DM2 and HV) underwent a detailed medical history and clinical neurological examination to confirm that they met the study inclusion/exclusion criteria. To evaluate the muscle strength of the hip extension, we used a manual muscle test rating with the MRC scale (range 0–5).

#### 2.2.2 Functional assessment of LEM

We evaluated both the strength and endurance of LEM. Maximal isometric lumbar extensor strength (MILEMS) (in kilograms) was examined in a sitting position using a MicroFET 2 handheld dynamometer (Hoggan Scientific, LLC.) and a purpose-designed chair. Each participant had five attempts, with 20 s to rest between attempts. The resulting muscle strength value was calculated as the mean of the second to fifth attempts. To evaluate LEM endurance (LEME), the Biering-Sørensen test was used. A detailed description of the examination methodology can be found in a previous study (22).

#### 2.2.3 MRI of lumbar spine and LPM

We used the 3 T Philips Ingenia MRI system with anterior and posterior receiving coils for the morphological evaluation. The examination included standard MRI sequences for lumbar spine assessment (turbo spin echo T2, T1, and STIR in the sagittal plane and T2 in the axial and coronal planes). Furthermore, we utilized a 6-point Dixon gradient echo sequence with multi-fat-peak compensation (seven) as well as eddy current correction (labeled mDixon Quant by the Philips company) for creating water, fat, in-phase and out-phase images, and fat fraction (FF) images with a resolution of 1.2*1.2*5 mm^3^ (23). The mDixon Quant sequence was acquired in the axial plane for the bilateral assessment of parameters of the LPM (multifidus muscle (MF) and erector spinae muscle (ES)) and psoas muscles (PS) (as a control muscle that is located in the lumbar region but is not a paraspinal muscle). The minimal sequence coverage was from intervertebral disk Th12/L1 to L5/S1. A detailed description of the MRI examination of the LPM, including the parameters of the MRI sequences, is covered in a previous article (21). All MRI images were assessed by an experienced radiologist to exclude any pathology within the study exclusion criteria.

**Figure 1:**
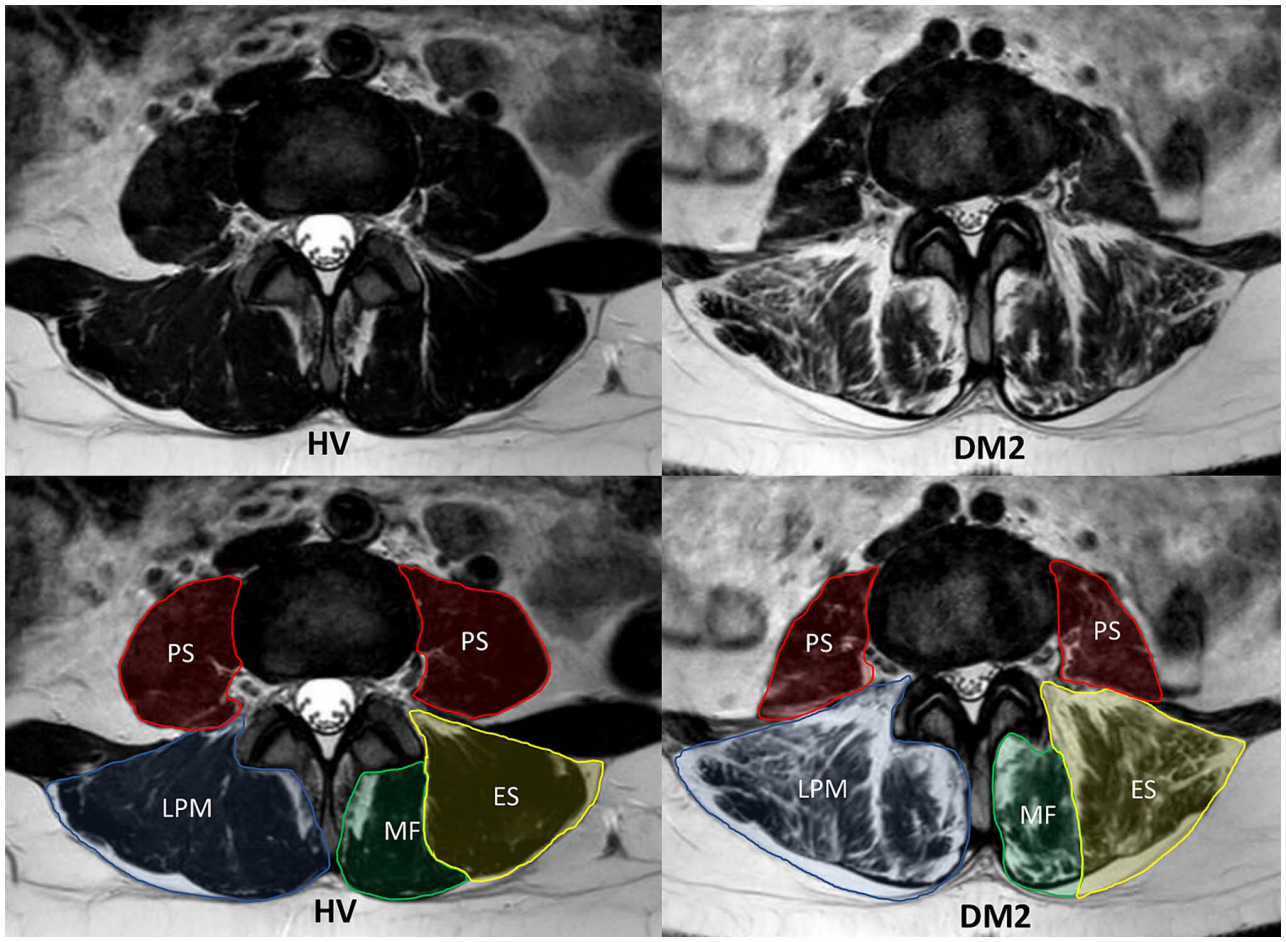
MRI T2-weighted sequence of the lumbar paraspinal muscles (LPM) and psoas muscles (PS) from a matched pair (patient with myotonic dystrophy type 2 (DM2) on the right and healthy volunteer (HV) on the left). The figures show MRI images in the axial plane at the same level of the intervertebral disk at L3/L4. In the lower figures, the individual segmented muscles are delineated. The LPM include the erector spinae muscle (ES) and the multifidus muscle (MF).

Image analysis of selected muscles (MF, ES, and PS bilaterally) was performed manually on all slices without any interpolation methods using the ITK-SNAP software application (24). Regions of interest were defined according to the recommendations of Crawford et al. (ES, MF) and Weinreb et al. (PS) (Figure 1) (25, 26).

All three muscles (MF, ES, and PS) were segmented primarily in the water image; the other images (fat, in-phase, and out-phase) were used for the detailed correction of the segmentation masks at all slices between the first axial MRI image above the superior endplate of L1 to the second axial MRI image below the inferior endplate of L5.

Segmentation masks of individual muscles were used to obtain muscle fat fraction (FF), total muscle volume (TMV), and functional muscle volume (FMV). The ES and MF were then combined bilaterally into a single muscle, LPM. The FF of each muscle was calculated as the average of FF in all muscle voxels bilaterally, expressed as a percentage. TMV for a given muscle was determined for each individual by summing the TMV values obtained from the right and left muscles. To estimate the FMV, we adopted the approach of Carlier et al. (10) and it was calculated as follows: FMV = TMV*(1 – FF). To evaluate muscle quality, defined as the ratio of muscle strength to muscle mass, we used the parameter MILEMS to FMV ratio (27–29).

### 2.3 Data analysis

For the statistical analysis, MatchIt, corrplot, and lme4 packages in R software (v 4.3.2) were utilized. All statistical tests and descriptive statistics respected the data type and distribution. Two-sided statistical tests were utilized, and statistical significance was set as *α* = 0.05.

DM2 patients and HV were matched to create 35 pairs based on propensity scores that considered age, sex, and BMI. To compare groups before matching, the Mann–Whitney U-test (continuous data) and Pearson’s Chi-squared test (categorical data) were used; after matching, the paired Wilcoxon signed rank test with continuity correction (continuous data) and McNemar’s Chi-squared test (categorical data) were used.

Of the qMRI parameters, FF and FMV were further analyzed as key biomarkers. To determine the correlation between qMRI parameters of LPM and functional parameters of LEM (MILEMS and LEME), Spearman correlation coefficients were calculated. Univariable generalized linear regression models with Gamma distribution and log link function were used to assess the effect of qMRI parameters on MILEMS and LEME, separately. Finally, to assess the evolution of functional parameters (MILEMS and LEME) and qMRI parameters with aging and to assess whether these changes differ between DM2 and HV, further multivariable generalized regression models with Gamma distribution and log link function were built with age, group, and their interaction as explanatory variables, and functional and qMRI parameters, separately, as dependent variables. In these models, the coefficients exp(beta) represent the percentage (not absolute) change in dependent variables. To assess the fitted model, the percentage of explained deviance out of the total deviance was calculated. Coefficients from the models are visualized using effects-plots.

## 3 Results

### 3.1 Basic characteristics of subjects

**Figure 2:**
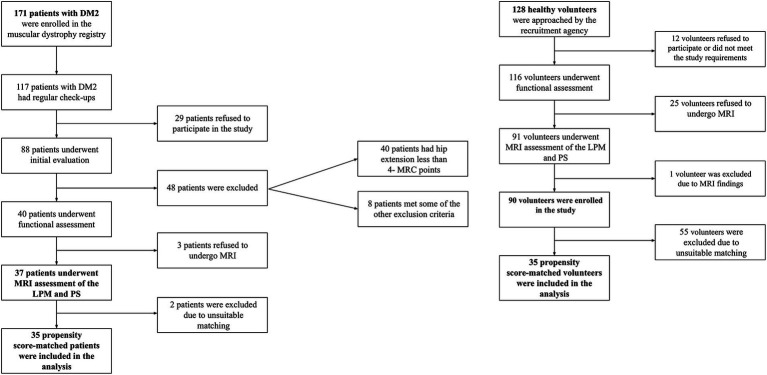
Study flowchart, including recruitment of patients with DM2 (myotonic dystrophy type 2) and healthy volunteers.

90 HV (45 men, 45 women) and 37 DM2 patients (12 men, 25 women, from 35 different DM2 families) were enrolled (Figure 2 – flowchart). The HV and DM2 groups differed significantly in age and height (Table 1). After matching, we had 35 pairs (HV-DM2) with corresponding physiological parameters (Table 1). Regarding the functional status of patients with DM2, 24 were able to stand up from a standardized 40-cm-high chair without any compensatory maneuver, 7 patients used a compensatory maneuver, 4 used one hand, and 2 used two hands to help them stand up. Only one used a walking aid.

**Table 1:** Basic characteristics of DM2 patients and HV before and after matching.

Before matching			
	HV N = 90	DM2 N = 37	*p*- value^2^
Age (years)^1^	37.5 (29.3; 48.0)	50.0 (41.0; 55.0)	**<0.001**
Sex (female)	45 (50.0%)	25 (67.6%)	0.071
Height (cm)^1^	175.0 (168.0; 182.8)	170.0 (164.0; 176.0)	**0.010**
Weight (kg)^1^	74.5 (63.0; 87.8)	71.0 (60.0; 92.0)	0.989
BMI (kg/m^2^)^1^	24.1 (21.7; 26.3)	24.9 (22.5; 29.4)	0.133

After matching			
	HV N = 35	DM2 N = 35	*p*- value^3^
Age (years)^1^	45.0 (38.0; 53.5)	50.0 (40.5; 54.0)	0.053
Sex (female)	23 (65.7%)	23 (65.7%)	
Height (cm)^1^	170.0 (165.0; 180.0)	170.0 (165.0; 176.0)	0.650
Weight (kg)^1^	75.0 (61.5; 89.0)	75.0 (60.0; 92.5)	>0.999
BMI (kg/m^2^)^1^	25.1 (22.1; 29.5)	25.8 (22.1; 30.3)	0.368

^1^Median (25%; 75%). ^2^Mann-Whitney U-test; Pearson’s Chi-squared test; ^3^Wilcoxon signed rank test with continuity correction; McNemar’s Chi-squared test. BMI, body mass index; HV, healthy volunteers; IQR, interquartile range; DM2, myotonic dystrophy type 2; N, number of subjects.

### 3.2 Functional and qMRI parameters of the LPM

Patients with DM2 had significantly lower MILEMS in a sitting position (*p* = 0.014) and LEME (*p* < 0.001) compared to HV. MRI parameters of LPM (combined MF and ES) showed significantly higher FF in patients with DM2 (*p* < 0.001), but the TMV and FMV of LPM were not significantly different from HV. The MILEMS to FMV ratio in LPM was significantly lower in DM2 patients compared to HV (*p* = 0.037; Table 2).

**Table 2:** Comparison of quantitative MRI and functional parameters in DM2 patients and HV before and after matching.

Before matching				
Parameters		HV N = 90^1^	DM2 N = 37^1^	*p*- value^2^
MILEMS – sitting position (kg)		49.7 (36.3; 67.3)	29.1 (21.8; 45.8)	**<0.001**
LEME [time(s)]		158.5 (123.5; 217.0)	53.0 (26.0; 123.0)	**<0.001**
Psoas muscle	Fat fraction (%)	6.1 (5.2; 7.1)	12.1 (9.6; 16.1)	**<0.001**
	Total muscle volume (cm^3^)	374 (272; 486)	222 (166; 276)	**<0.001**
	Functional muscle volume (cm^3^)	342 (248; 452)	179 (130; 248)	**<0.001**
Lumbar paraspinal muscles	Fat fraction (%)	8.9 (6.2; 12.1)	22.5 (16.4; 28.6)	**<0.001**
	Total muscle volume (cm^3^)	763.0 (620.4; 933.7)	724.7 (644.1; 888.0)	0.960
	Functional muscle volume (cm^3^)	661.1 (536.4; 841.4)	580.0 (491.1; 658.6)	**0.009**
MILEMS to FMV ratio – sitting position (kg/cm^3^)		0.076 (0.060; 0.097)	0.054 (0.042; 0.068)	**<0.001**

After matching				
		HV N = 35^1^	DM2 N = 35^1^	*p*- value^3^
MILEMS – sitting position (kg)		39.7 (31.5; 64.0)	29.5 (21.9; 45.9)	**0.014**
LEME [time(s)]		148.0 (111.5; 219.5)	64.0 (27.0; 125.5)	**<0.001**
Psoas muscle	Fat fraction (%)	6.9 (6.0; 9.0)	12.1 (9.7; 15.7)	**<0.001**
	Total muscle volume (cm^3^)	288 (232; 423)	225 (167; 285)	**<0.001**
	Functional muscle volume (cm^3^)	272 (218; 389)	193 (134; 253)	**<0.001**
Lumbar paraspinal muscles	Fat fraction (%)	11.3 (8.7; 16.2)	21.3 (14.7; 27.9)	**<0.001**
	Total muscle volume (cm^3^)	671.0 (571.9; 872.5)	739.5 (646.7; 890.5)	0.179
	Functional muscle volume (cm^3^)	567.9 (485.2; 726.6)	580.5 (513.1; 670.9)	0.334
MILEMS to FMV ratio – sitting position (kg/cm^3^)		0.074 (0.049; 0.097)	0.054 (0.043; 0.071)	**0.037**

^1^Median (25%; 75%). ^2^Mann-Whitney U-test. ^3^Wilcoxon signed rank test with continuity correction. DM2, myotonic dystrophy type 2; HV, healthy volunteers; LEME, lumbar extensor muscle endurance; MILEMS, maximal isometric lumbar extensor muscle strength; N, number of subjects.

The control muscle (PS) showed higher FF (*p* < 0.001) and lower TMV and FMV (*p* < 0.001) in the DM2 patients compared to HV.

The qMRI parameters of MF and ES are shown in Supplementary Table S1.

### 3.3 Relationship between qMRI parameters of LPM and functional parameters in patients with DM2

**Figure 3:**
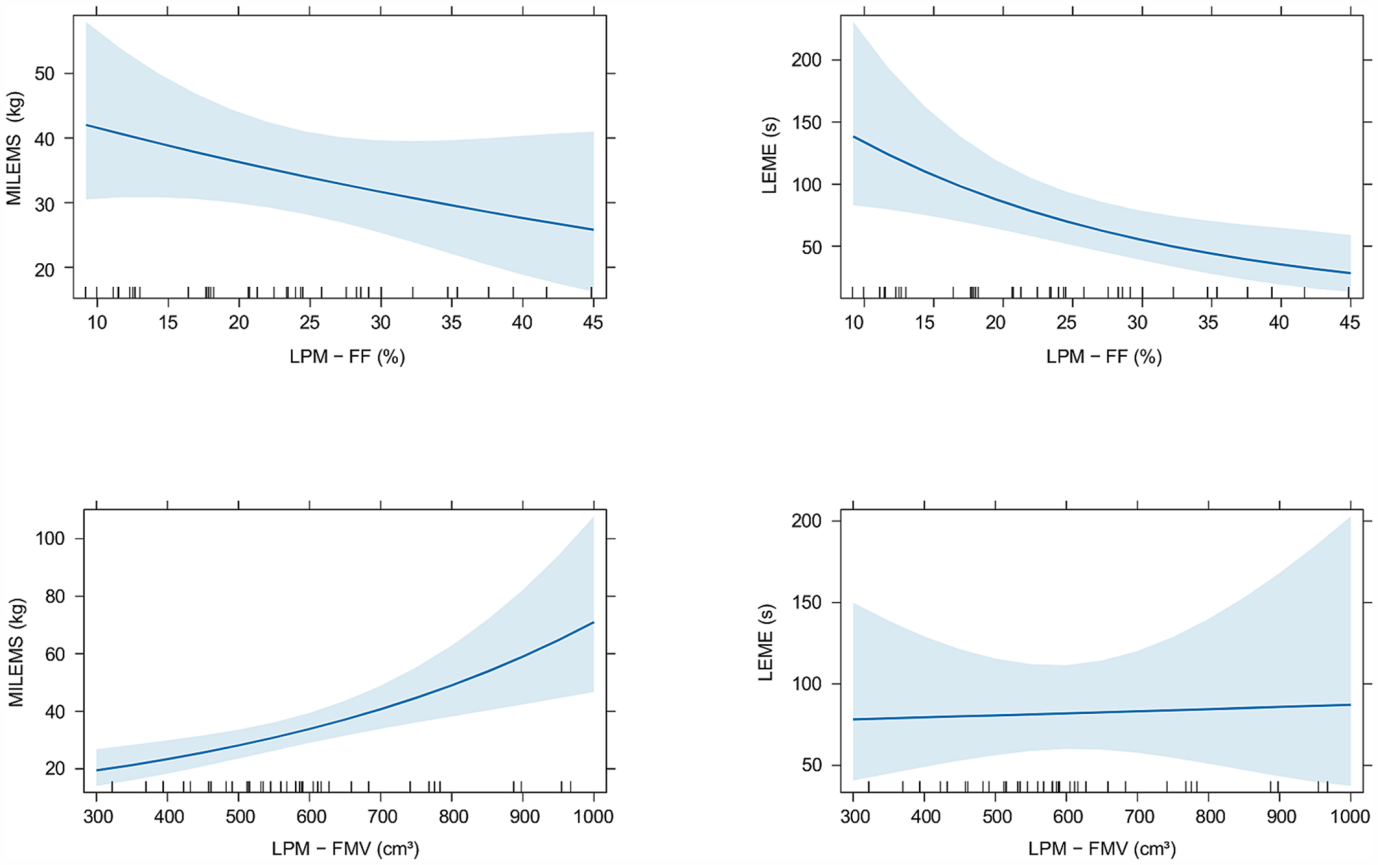
Graphs showing the relationship of quantitative MRI parameters (fat fraction (FF) and functional muscle volume (FMV)) of the lumbar paraspinal muscles (LPM) and functional parameters (maximal isometric lumbar extensor muscle strength (MILEMS) and lumbar extensor muscle endurance (LEME)) in patients with DM2. Estimated means with corresponding 95% confidence intervals are shown.

A statistically significant correlation was found between the FMV of LPM and MILEMS (ρ_Spearman_ = 0.5; CI_95%_ (0.21; 0.72); *p* = 0.001) and between the FF of LPM and LEME (ρ_Spearman_ = −0.49; CI_95%_ (−0.70; −0.18); *p* = 0.002). The regression models showed that each 100 cm^3^ increase in the FMV of LPM is associated with a significant increase in MILEMS by an average of 20% (*p* < 0.001) and each 10% increase in the FF of LPM is associated with a significant decrease in LEME by an average of 36% (*p* = 0.006; Table 3; Figure 3). There was also a decrease in MILEMS with increasing FF of LPM and a slight increase in LEME with increasing FMV of LPM, but these changes were not significant in either case (Table 3; Figure 3).

**Table 3:** Univariable generalized linear regression models for functional parameters of lumbar extensor muscles in DM2.

	Exp (Beta)	95% CI^1^	*p*- value	Explained deviance (%)
Models for MILEMS				
Lumbar paraspinal muscles – fat fraction (per 10%)	0.87	0.72–1.06	0.160	5.3
Lumbar paraspinal muscles – functional muscle volume (per 100 cm^3^)	1.20	1.10–1.32	**<0.001**	31.7
Models for LEME				
Lumbar paraspinal muscles – fat fraction (per 10%)	0.64	0.49–0.86	**0.006**	12.2
Lumbar paraspinal muscles – functional muscle volume (per 100 cm^3^)	1.02	0.86–1.21	0.872	0.1

^1CI, Confidence Interval; LEME, lumbar extensor muscle endurance; MILEMS, maximal isometric lumbar extensor muscle strength. Values in bold are statistically significant.^

### 3.4 Evolution of functional and qMRI parameters of LPM with aging

**Figure 4:**
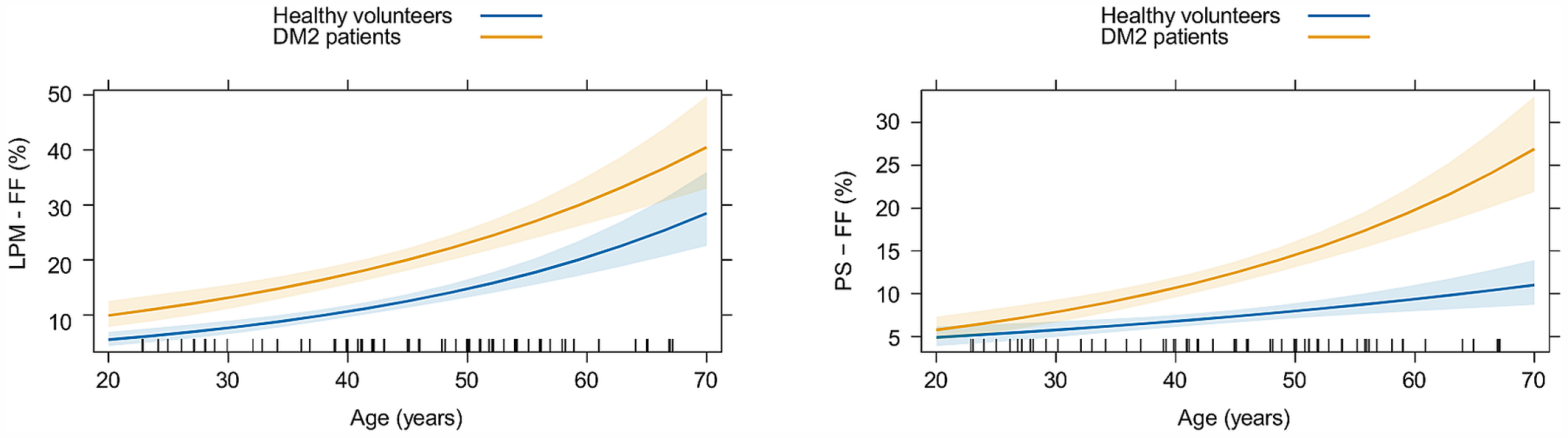
Graphs showing the evolution of the fat fraction (FF) of lumbar paraspinal muscles (LPM) and psoas muscle (PS) with aging in matched healthy volunteers (in blue) and patients with myotonic dystrophy type 2 (in yellow). Estimated means with corresponding 95% confidence intervals are shown.

When comparing the evolution of functional parameters (MILEMS and LEME) in DM2 patients to HV with aging, we found that the evolution of MILEMS and LEME with aging did not significantly differ between DM2 and HV, and aging has no statistically significant effect on functional parameters in either group (Supplementary Table S2; Supplementary Figure S1). Regarding qMRI parameters, DM2 patients and HV did not significantly differ in the evolution of the FMV of LPM with aging (Supplementary Table S3; Supplementary Figure S2) and the FMV did not change significantly with age in either group. The FF of LPM increased significantly in both groups with age (*p* < 0.001); there was no significant difference in the rate of its increase with aging between DM2 and HV (Table 4; Figure 4). Thus, there was no significant additional functional or morphological deterioration of LPM over time in DM2 patients compared to HV. In PS, there was a significant increase in FF in both groups (*p* < 0.001) with aging, but unlike LPM, DM2 patients had a significantly faster increase in FF over time compared to HV (*p* = 0.014). Specifically, DM2 patients had a 16% greater increase in the FF of PS per every 10 years compared to HV (Table 4; Figure 4). The FMV of PS decreased non-significantly in both groups with aging and there was no significant difference between the two groups (Supplementary Table S3; Supplementary Figure S2).

**Table 4:** Multivariable generalized linear regression models for muscle fat fraction with aging.

	Exp (Beta)	95% CI^1^	*p*- value
Model for lumbar paraspinal muscles – fat fraction (%)^2^			
(Intercept)	0.29	0.20–0.42	**<0.001**
**Group**			
Healthy volunteers	—	—	
DM2 patients	1.98	1.16–3.36	**0.015**
Age (per 10 years)	1.39	1.28–1.50	**<0.001**
Age (per 10 years) * DM2 patients	0.95	0.85–1.07	0.418
^2^Explained deviance: 72.5%			
Model for psoas muscle – fat fraction (%)^3^			
(Intercept)	3.58	2.45–5.29	**<0.001**
**Group**			
Healthy volunteers	—	—	
DM2 patients	0.88	0.51–1.50	0.632
Age (per 10 years)	1.17	1.08–1.28	**<0.001**
Age (per 10 years) * DM2 patients	1.16	1.03–1.30	**0.014**
^3^Explained deviance: 72.3%.			

^1CI, Confidence Interval. DM2, myotonic dystrophy type 2.^

## 4 Discussion

This study focuses on the evaluation of qMRI parameters of the LPM using an advanced MRI method (6-point Dixon gradient echo sequence) in patients with DM2, their correlation with functional examination, and the evolution of these parameters with aging. DM2 patients showed increased FF of LPM compared to matched HV. The FMV of LPM correlated significantly with strength (MILEMS), and the FF of LPM correlated significantly with endurance (LEME); however, these radiological parameters cannot be used as reliable biomarkers of LEM functional impairment in DM2 patients as the correlation coefficients and variability of MILEMS and LEME explained by radiological parameters were relatively low. Further, there was no significant additional worsening of the functional and morphological parameters of LPM in DM2 patients with increasing age compared to HV; therefore these parameters cannot be considered as biomarkers of disease progression.

To the best of our knowledge, qMRI parameters of LPM in DM2 have not yet been evaluated and published; only Kornblum et al. described frequent fatty degeneration of the ES in DM2, graded semi-quantitatively by the Mercuri scale on whole-body MRI (14). In our study, all the muscles evaluated showed higher FF, a typical finding that has been described in different types of muscular dystrophies, including DM2 (11–13, 30). The muscle volumes (TMV and FMV) of LPM were not significantly different in DM2 patients compared to HV, but the muscle volumes of PS were significantly smaller. This distinctive behavior of LPM volumes compared to PS volumes can be explained by the different representation of muscle fibers in individual muscles. A typical feature of LPM is the predominance of slow-twitch fibers (type 1); while PS has a predominance of fast-twitch fibers (type 2) (31, 32). In DM2 patients, mainly the atrophy of type 2 fibers is described (33–35). The literature describes that the fat infiltration of skeletal muscles often occurs without loss of muscle volume (30). It has also been shown that the onset and progression of changes in muscle volume and fatty infiltration are distinct, independent pathological processes differing between muscle groups in patients with muscular dystrophy (36).

Dahlquist et al. mentioned parameter muscle contractility (strength per fat-free cross-sectional area), that is usually reduced in muscular dystrophies and reflects the quality of the muscle composition (11). In our study, we used the parameter MILEMS to FMV ratio as FMV better reflects contractile muscle size than a fat-free (lean) cross-sectional area. We have shown that DM2 patients have a significantly lower MILEMS to FMV ratio in the LPM, which can be interpreted as 1 cm^3^ of fat-free LPM generates less strength in DM2 patients compared to HV. This indicates reduced muscle quality in DM2.

Dahlquist et al. described that muscle fat content negatively correlated with muscle function in neuromuscular diseases and observed that changes in fat content precede changes in function (11). Far fewer studies have evaluated the relationship between strength and muscle volume, most likely because muscle volume is more difficult to determine than FF, requiring manual muscle segmentation or a sophisticated automatic segmentation method using artificial intelligence. The literature suggests that muscle strength correlates more strongly with FMV than with FF (10). This is supported by our study in which MILEMS correlated with the FMV of LPM, whereas MILEMS showed only a trend to decrease with increasing FF, which was not statistically significant. In contrast, LEME correlated more strongly with FF compared to the FMV of LPM in DM2.

The relationship between functional and qMRI parameters is complicated and discordance was reported in DM2 patients (13, 14). There are numerous possible explanations. First, fatty degeneration may not always result in a loss of muscle strength or a decrease in functional status (8). Second, in addition to fat replacement in muscular dystrophies, muscle mass is replaced by connective tissue, directly impacting the muscle function. Fibrosis is often distributed around muscle fibers and its imaging is very challenging (10, 11). As fibrous tissue is composed mainly of collagen fibers, which appear as non-fat tissue on 6-point Dixon chemical shift imaging, making it indistinguishable from muscle, it may be included in the FMV and significantly influence FMV-strength correlations. Different imaging protocols have been suggested for its differentiation, particularly gadolinium contrast enhanced imaging, which is widely used in myocardial imaging. However, the need to apply a contrast agent is a disadvantage here; moreover, dystrophic muscle fibers in neuromuscular disorders could lead to the contrast agent penetrating into the muscle itself (10). Therefore, gadolinium contrast enhanced imaging is not suitable for fibrosis imaging in neuromuscular disorders. More advantageous seem to be methods using ultra-short echo times or magnetic resonance elastography (10, 30, 37). Last but not least, errors in muscle segmentation and/or MILEMS examination can occur, even though we proved these methods to be reliable in previous studies (21, 22). It should be noted that MILEMS and LEME are not the result of isolated LPM or LEM activity, but can be influenced by the involvement of other muscle groups.

Madrid et al. showed that in DM2 patients, the FF of the affected muscles of the lower extremities correlated with motor performance. The FF of the lower extremity muscles was thus considered as a potential biomarker of disease severity (12). In our study, despite the demonstrated statistically significant correlation between FMV and MILEMS and FF and LEME, we cannot consider qMRI parameters as reliable biomarkers of LPM impairment as the confidence intervals were relatively wide and correlation coefficients and variability of functional parameters explained by FF and FMV were relatively low. The discrepancy with the Madrid et al. study is probably due to the different selection of muscles analyzed (LPM vs. leg muscles) (12).

It is reported that symptoms such as muscle weakness and myotonia worsen progressively with increasing age in DM2 (38). Peric et al. (13) analyzed the FF of leg muscles in DM2 patients in relation to different disease durations; we do not consider this method accurate. The definition of disease duration in DM2 patients is questionable. Disease duration can refer to either the duration of symptoms, which is an anamnestic parameter that can be very imprecise, or the duration of the disease since genetic confirmation of the diagnosis, which is not reflective of the disease duration because there is often a significant diagnostic delay (Peric et al. reported diagnostic delays of 12.4 ± 10.9 years) (13). To assess the progression of LPM impairment in patients with DM2 over time, we compared the evolution of LPM parameters with aging between patients and HV. This approach was selected because muscle parameters change with age even in healthy people, with a significant increase in FF with aging, as shown in this study. We did not find a significant difference in the rate of deterioration in functional parameters (MILEMS, LEME) or MRI parameters (FF, FMV) of the LPM in patients with DM2 compared to HV. On the other hand, DM2 patients showed a faster increase in the FF of PS with age compared to HV. This suggests more pronounced radiological and probably also functional worsening of PS impairment in DM2 with age. It can be concluded that the evolution of LPM impairment in DM2 patients does not significantly differ from natural age-related structural and functional changes. Therefore, qMRI parameters of LPM and/or changes in MILEMS and LEME cannot be used as a biomarker of DM2 progression. On the other hand, the FF of PS showed an additional progressive increase with age in DM2 patients. This probably reflects disease progression, but it would require further analysis and correlation with functional muscle testing of PS, which was not the focus of this study. The different behavior of the LPM and PS during aging in DM2 probably arises again from different muscle fiber representation in these muscles and the atrophy of predominantly type 2 fibers in DM2 (33–35).

This study has limitations. Most importantly, as DM2 is an orphan disease, the number of patients is limited. This may affect the power of statistical tests and lead to greater sensitivity to individuals with outlier parameters. However, this study evaluated muscle MRI from the largest number of DM2 patients – the number of patients in previously reported studies with DM2 was only as high as 14 (12–15). Second, the exclusion criteria led to the recruitment of patients with only mild functional impairment. Also, the maximum age of our DM2 group subjects was 67 years, as older patients failed to meet these strict criteria. Therefore, conclusions cannot be generalized for the whole population of patients with DM2, but rather for a selected cohort of young to young-old patients with mild DM2.

This is the first study evaluating in detail the LPM in DM2 patients, which has been neglected and only marginally mentioned in the literature, despite the importance of these muscles in maintaining upright posture and spinal health. Further, functional and qMRI parameters of the LPM of DM2 patients were compared with a cohort of HV of the same sex and similar age, weight, and height. This approach is very important because the morphological and functional parameters of the muscles vary significantly with the physiological parameters of the individual. The contribution of this study also lies in the chosen methodology of the MRI examination. In order to obtain the most accurate qMRI parameters, we used a 6-point Dixon gradient echo sequence and performed segmentation of ES, MF, and PS over the entire muscle, although it was time consuming.

This study supports the classification of DM2 as an axial myopathy; this should be considered when managing patients. We believe that patients may benefit from physiotherapy focused on strengthening the LEM and improving the coordination of the deep stabilization system. A well-chosen exercise program may lead to a reduction in low back pain, for which LEM dysfunction is a risk factor (7). LPM dysfunction does not evince additional progressive worsening with aging in patients with DM2 compared to HV. Therefore, camptocormia and neuromuscular scoliosis will not be encountered in these patients or only very rarely (39); this is in line with common clinical practice. As DM2 is an under-diagnosed condition, we recommend keeping it in mind in the differential diagnosis of patients with more extensive LPM fatty infiltration on MRI than would be expected given the patient’s age and sex. This is particularly relevant in the evaluation of lumbar spine MRI in patients with chronic low back pain. Further studies using advanced MRI are needed to evaluate fibrosis as a potential biomarker of DM2 severity and progression.

## Data Availability

The raw data supporting the conclusions of this article will be made available by the authors, without undue reservation.
