# Supplementary material for: Quantitative magnetic resonance imaging parameters of lumbar paraspinal muscle impairment in myotonic dystrophy type 2 and their evolution with aging
Source: Front Neurol. 2025 Feb 19;16:1525952. doi: 10.3389/fneur.2025.1525952 (PMC11879826; doi:10.3389/fneur.2025.1525952)
Supplement: Supplementary file 1 [file Table_1.docx]

Supplementary Material

# Supplementary Figures and Tables

## Supplementary Figures

**Supplementary Figure 1.** Graphs showing the evolution of the maximal isometric lumbar extensor muscle strength (MILEMS) and lumbar extensor muscle endurance (LEME) with ageing in matched healthy volunteers (in blue) and patients with myotonic dystrophy type 2 (in yellow). Estimated means with corresponding 95% confidence intervals are shown.

**Supplementary Figure 2.** Graphs showing the evolution of the functional muscle volume (FMV) of lumbar paraspinal muscles (LPM) and psoas muscle (PS) with ageing in matched healthy volunteers (in blue) and patients with myotonic dystrophy type 2 (in yellow). Estimated means with corresponding 95% confidence intervals are shown.

## Supplementary Tables

**Supplementary Table 1.** Comparison of quantitative MRI parameters of the erector spinae muscle and the multifidus muscle in DM2 patients and HV before and after matching.

| **Before matching** | | | | |
| --- | --- | --- | --- | --- |
| **Parameter** |  | **HV**  N=90^1^ | **DM2**  N=37^1^ | **p-value**^2^ |
| **Erector spinae muscle** | **Fat fraction (%)** | 8.3 (5.8; 10.4) | 20.2 (13.7; 27.3) | **<0.001** |
|  | **Total muscle volume (cm³)** | 555 (430; 701) | 529 (473; 639) | 0.817 |
|  | **Functional muscle volume (cm³)** | 478 (385; 610) | 422 (369; 493) | **0.020** |
| **Multifidus muscle** | **Fat fraction (%)** | 11.1 (7.0; 16.7) | 26.5 (18.9; 32.4) | **<0.001** |
|  | **Total muscle volume (cm³)** | 211 (184; 244) | 201 (171; 235) | 0.507 |
|  | **Functional muscle volume (cm³)** | 184 (153; 211) | 156 (126; 189) | **0.001** |
| **After matching** | | | | |
|  |  | **HV**  N=35^1^ | **DM2**  N=35^1^ | **p-value**^3^ |
| **Erector spinae muscle** | **Fat fraction (%)** | 9.1 (8.2; 14.0) | 20.0 (13.5; 26.5) | **<0.001** |
|  | **Total muscle volume (cm³)** | 450 (405; 666) | 529 (478; 651) | 0.069 |
|  | **Functional muscle volume (cm³)** | 404 (348; 540) | 422 (376; 493) | 0.544 |
| **Multifidus muscle** | **Fat fraction (%)** | 15.8 (10.4; 21.1) | 25.9 (18.3; 31.8) | **<0.001** |
|  | **Total muscle volume (cm³)** | 207 (173; 245) | 203 (175; 236) | 0.961 |
|  | **Functional muscle volume (cm³)** | 165 (145; 201) | 156 (128; 191) | 0.131 |
| ^1^Median (25%; 75%)  ^2^Mann-Whitney *U*-test  ^3^Wilcoxon signed rank test with continuity correction  BMI – body mass index, DM2 – myotonic dystrophy type 2, HV – healthy volunteers, N – number of subjects | | | | |

**Supplementary Table 2.** Multivariable generalised linear regression models for functional parameters with ageing.

|  | **Exp (Beta)** | **95% CI**^1^ | **p-value** |
| --- | --- | --- | --- |
|  | **Model for MILEMS (kg)^2^** | | |
| **(Intercept)** | 59.6 | 30.4 – 121 | **<0.001** |
| **Group** |  |  |  |
| Healthy volunteers | — | — |  |
| DM2 patients | 0.77 | 0.30 – 2.00 | 0.593 |
| **Age (per 10 years)** | 0.95 | 0.82 – 1.10 | 0.478 |
| **Age (per 10 years) * DM2 patients** | 1.00 | 0.82 – 1.23 | 0.994 |
| ^2^Explained deviance: 8.5% | | | |
|  | **Model for LEME (time(s))^3^** | | |
| **(Intercept)** | 170 | 61.4 – 515 | **<0.001** |
| **Group** |  |  |  |
| Healthy volunteers | — | — |  |
| DM2 patients | 1.52 | 0.34 – 6.88 | 0.585 |
| **Age (per 10 years)** | 1.00 | 0.79 – 1.26 | 0.993 |
| **Age (per 10 years) * DM2 patients** | 0.78 | 0.57 – 1.08 | 0.137 |
| ^3^Explained deviance: 16.4% | | | |
| ^1^CI = Confidence Interval  DM2 – myotonic dystrophy type 2, LEME – lumbar extensor muscle endurance, MILEMS – maximal isometric lumbar extensor muscle strength | | | |

**Supplementary Table 3.** Multivariable generalised linear regression models for functional muscle volume with ageing.

|  | **Exp (Beta)** | **95% CI**^1^ | **p-value** |
| --- | --- | --- | --- |
|  | **Model for lumbar paraspinal muscles – functional muscle volume (cm³)**^2^ | | |
| **(Intercept)** | 8.33 | 5.59 – 12.5 | **<0.001** |
| **Group** |  |  |  |
| Healthy volunteers | — | — |  |
| DM2 patients | 0.90 | 0.51 – 1.58 | 0.706 |
| **Age (per 10 years)** | 0.94 | 0.86 – 1.03 | 0.180 |
| **Age (per 10 years) * DM2 patients** | 1.01 | 0.90 – 1.14 | 0.838 |
| ^2^Explained deviance: 5.8% | | | |
|  | **Model for psoas muscle – functional muscle volume (cm³)**^3^ | | |
| **(Intercept)** | 491 | 299 – 820 | **<0.001** |
| **Group** |  |  |  |
| Healthy volunteers | — | — |  |
| DM2 patients | 1.04 | 0.51 – 2.09 | 0.921 |
| **Age (per 10 years)** | 0.90 | 0.81 – 1.01 | 0.065 |
| **Age (per 10 years) * DM2 patients** | 0.91 | 0.79 – 1.06 | 0.232 |
| ^3^Explained deviance: 36.6% | | | |
| ^1^CI = Confidence Interval  DM2 – myotonic dystrophy type 2 | | | |
