# Supplementary figures and images for: Quantitative magnetic resonance imaging parameters of lumbar paraspinal muscle impairment in myotonic dystrophy type 2 and their evolution with aging
Source: Front Neurol. 2025 Feb 19;16:1525952. doi: 10.3389/fneur.2025.1525952 (PMC11879826; doi:10.3389/fneur.2025.1525952)

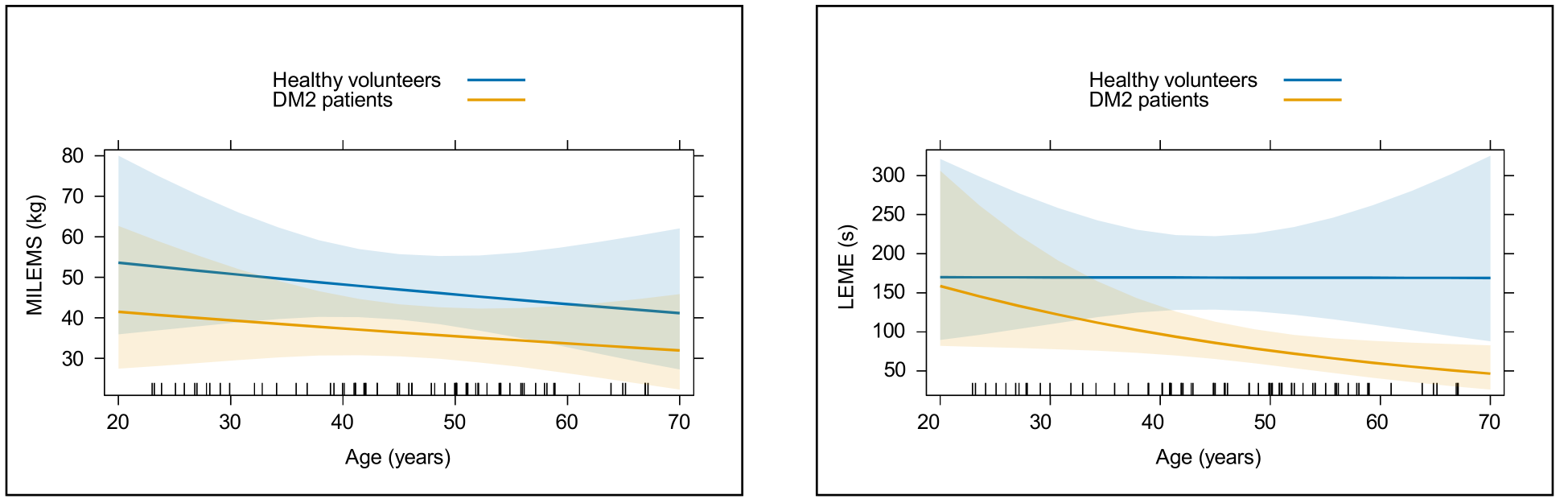

Supplement: Supplementary file 2 [file Image_1.tiff]

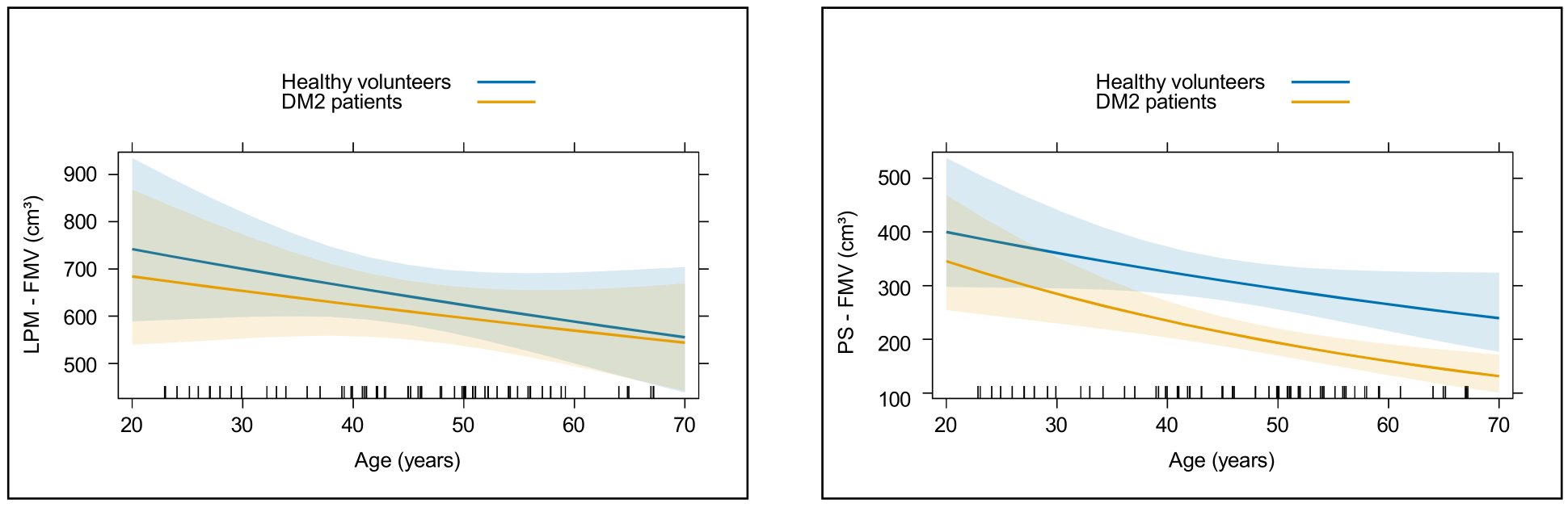

Supplement: Supplementary file 3 [file Image_2.tiff]
